# Polygenic risk scores in pharmacogenomics: methodological challenges, current applications, and perspectives for clinical implementation

**DOI:** 10.1515/medgen-2026-3013

**Published:** 2026-07-08

**Authors:** Carina M. Mathey, Martin Coenen, Ingolf Cascorbi, Per Hoffmann

**Affiliations:** University Hospital Bonn Institute of Human Genetics Venusberg-Campus 1, Gebäude 76 53127 Bonn Germany; University Hospital Bonn Phase I-Unit Venusberg-Campus 1 53127 Bonn Germany; University Hospital Schleswig-Holstein Institute of Experimental and Clinical Pharmacology Arnold-Heller-Str. 3 24105 Kiel Germany; University Hospital Bonn Institute of Human Genetics Venusberg-Campus 1, Gebäude 76 53127 Bonn Germany

**Keywords:** Pharmacogenetics, pharmacogenomics, polygenic risk score, precision medicine, clinical translation

## Abstract

Inter-individual variability in drug response remains a major challenge in clinical pharmacology. While clinical pharmacogenetics is largely based on actionable single-gene variants, increasing GWAS evidence indicates that many drug-response phenotypes are polygenic. Polygenic risk scores (PRS) offer a framework to capture cumulative effects across pharmacokinetic, pharmacodynamic, and disease-related pathways, enabeling probabilistic risk stratification beyond monogenic models.

This review summarizes the current status of PRS in pharmacogenomics (PGx-PRS), highlighting key challenges including limited sample sizes of drug-exposed cohorts, heterogeneous phenotype definitions, ancestry-related portability issues, and incomplete representation of complex pharmacogenes such as *CPY2D6*. Current applications are discussed across cardiovascular, psychiatric, and oncological settings, where PRS show emerging potential for benefit-risk stratification but remain insufficiently validated for routine care.

Clinical implementation will require standardized methodologies multi-ancestry validation, integration with rare-high impact ADME (Absorption, Distribution, Metabolism, and Excretion) variants, and clearer regulatory pathways. With these advances, PGx-PRS may become clinically relevant tools for precision prescribing and drug development.

## Introduction

Inter-individual variability in drug response remains a persistent and clinically significant challenge in pharmacology, as patients receiving identical medications and doses frequently exhibit divergent outcomes with respect to both therapeutic efficacy and safety [1]. To date, clinical pharmacogenetics has been predominantly grounded in well-characterized single-gene–drug interactions – most prominently involving cytochrome P450 (CYP) enzymes or other phase I or II enzymes such as the thiopurine S-methyltransferase (*TPMT*) – for which actionable prescribing recommendations have been established by expert consortia such as the Dutch Pharmacogenomics Working Group (DPWG) and the Clinical Pharmacogenetics Implementation Consortium (CPIC) [2 – 6].

Paradigmatic examples of such monogenic associations with established clinical relevance include dihydropyrimidine dehydrogenase (*DPYD*) variants predicting severe fluoropyrimidine toxicity and the pharmacogenomic interaction between irinotecan or atazanavir and *UGT1A1* polymorphisms causing neutropenia or affecting bilirubin metabolism [7 – 10]. These examples illustrate how specific germline variants in drug-metabolizing enzymes or transporters can confer substantial and predictable changes in drug disposition or toxicity risk, thereby enabling genotype-guided prescription within defined gene–drug pairs. For a comprehensive overview see Lauschke & Ingelman-Sundberg 2026 [11].

**Figure 1:**
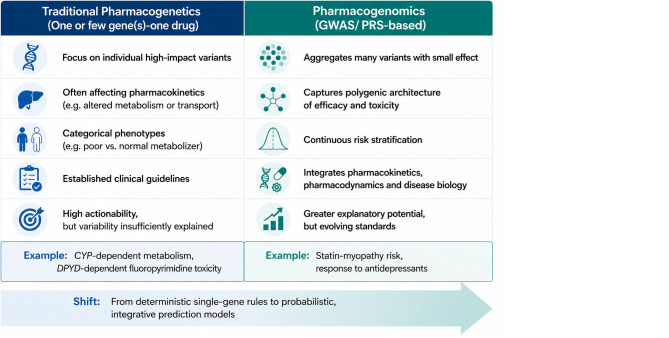
Pharmacogenetics vs. pharmacogenomics.

While these monogenic associations have demonstrated clear clinical utility, they collectively account for only a fraction of the observed inter-individual variability in drug response. Increasing evidence from genome-wide association studies (GWAS) indicates that numerous pharmacological phenotypes – including treatment efficacy, dose requirements, and susceptibility to adverse drug reactions – exhibit a polygenic architecture shaped by the cumulative effects of many genetic variants of individually small effect, acting across pharmacokinetic, pharmacodynamic, and disease-related pathways [12, 13] (Figure 1). Accordingly, this review focuses primarily on common-variant, GWAS-based approaches.

Classical examples include the genetic determinants of warfarin dose variability, where variants in *VKORC1* and *CYP2C9* explain a substantial proportion of inter-individual variation but additional loci contribute to residual variability [14, 15]. However, despite this biological rational and genetic associations, prospective randomized controlled trials evaluating genotype-guided dosing have produced inconsistent results, and clinical implementation remains limited and geographically variable. This illustrates that even well-established genetic-predictors do not necessarily translate directly into routine clinical practice. Similarly, GWAS have identified multiple loci influencing clopidogrel response and platelet reactivity, extending beyond the well-known *CYP2C19* variants [16]. For adverse drug reactions, genome-wide analyses of statin-associated myopathy have revealed variants in *SLCO1B1* as major contributors while suggesting additional polygenic influences on toxicity risk [17, 18]. In psychiatric pharmacotherapy, large-scale studies of antidepressant treatment response and antipsychotic efficacy likewise demonstrate highly polygenic architectures with individually modest effect sizes distributed across numerous loci [19 – 21]. Collectively, these findings provide a conceptual rationale for moving beyond single-variant pharmacogenetic models toward polygenic frameworks capable of capturing the broader genomic contribution to drug response variability.

Accordingly, there is a growing scientific and clinical need to extend pharmacogenetic testing beyond single-gene paradigms and to advance the development and implementation of polygenic risk score (PRS)-based strategies tailored specifically to pharmacological phenotypes [22]. However, several methodological, scientific, and clinical challenges remain to be addressed before such approaches can be translated into routine clinical application. Against this background, the present article provides (i) a critical overview of the methodological challenges inherent to PRS development in the context of pharmacogenomics, (ii) a synthesis of current applications of PRS across therapeutic areas, and (iii) a framework for the requirements underlying responsible integration into routine clinical care.

## Methodological challenges in PRS development in the context of pharmacogenomics

The development of PRS for pharmacogenomics applications involves distinct methodological challenges compared to disease-oriented PRS, most notably with respect to the selection and interpretation of appropriate data sources. A central issue is the frequent reliance on disease-derived GWAS summary statistics as an indirect proxy for pharmacological phenotypes. Although such datasets offer large sample sizes – often exceeding one million participants – and correspondingly robust statistical power, the extrapolation of disease-associated variants may systematically overlook drug-specific genetic effects that are mechanistically unrelated to disease susceptibility per se. For example, a coronary artery disease (CAD) PRS does not necessarily capture the genetic determinants of statin efficacy or statin-induced adverse effects, which are determined more by pathways of lipid metabolism, hepatic transport, and skeletal muscle toxicity than by the risk of atherosclerotic disease itself [23, 24]. Similarly, a genetic predisposition to schizophrenia is not equivalent to predictors of response to clozapine treatment, in which immune, metabolic, and drug-metabolism pathways play a central role – pathways that differ mechanistically from the underlying cause of disease [25]. These examples illustrate the fundamental conceptual distinction between gene–disease associations and gene–drug interactions.

A further critical limitation concerns the weighting of variant effect sizes: estimates derived from disease GWAS may not translate to pharmacological outcomes, particularly when treatment response reflects a composite of pharmacokinetic, pharmacodynamic, and disease-modifying biological processes. Although direct pharmacogenomic GWAS conducted in drug-exposed cohorts represent a more analytically specific alternative they remain comparatively underpowered due to limited sample sizes, heterogeneous phenotype definitions, and practical challenges in capturing adherence patterns, dosing history, and co-medication effects across study populations [26, 27].

Population structure introduces additional complexity into PRS construction. Differences in linkage disequilibrium (LD) patterns between ancestral groups can result in the tagging of non-causal proxy variants rather than true causal loci, and the pronounced overrepresentation of individuals of European-ancestry – often exceeding 80 % in many datasets and frequently even higher in pharmacogenomic GWAS cohorts – substantially limits PRS portability and predictive accuracy in genetically diverse populations [28].

An additional limitation of particular relevance to pharmacogenomics is the inadequate representation of structurally complex pharmacogenes within GWAS-derived datasets. Key pharmacokinetic loci such as *CYP2D6* – encoding the most polymorphic drug-metabolizing enzyme in humans – exhibit copy number variation, hybrid gene arrangements, and haplotype-level star-allele diversity that are not reliably detected by conventional SNP microarrays or accurately inferred through LD-based imputation, thereby leading to the systematic underrepresentation of major pharmacokinetic determinants within conventional PRS frameworks [29, 30].

Addressing this technical gap necessitates the integration of complementary genotyping and sequencing strategies. Conventional microarrays, while cost-effective and scalable for genome-wide discovery, are insufficient as the sole genotyping strategy for pharmacogenomic PRS which require accurate representation of complex ADME (Absorption, Distribution, Metabolism, and Exrection) genes. Short-read next-generation sequencing (NGS) enables higher-resolution variant detection than microarrays and has improved the pharmacogenetic characterization of structurally complex loci; however, it remains limited in its ability to resolve repetitive regions and ambiguous haplotype phasing. Long-read sequencing technologies, such as those provided by Pacific Biosciences (PacBio) and Oxford Nanopore Technologies (ONT), enable substantially improved resolution of structural variants, copy number variation, and phased haplotypes, thereby enabling more comprehensive characterization of complex pharmacogenetic loci such as *CYP2D6* [31 – 33]. Integrating long-read sequencing data with genome-wide association approaches may therefore represent a methodological prerequisite for clinically meaningful polygenic pharmacogenomic models.

Finally, the choice of PRS construction methodology must account for the hybrid genetic architecture of drug response. Conventional disease-oriented algorithms such as LDpred2 [34] or PRS-CS [35] assume highly polygenic architectures without incorporating pharmacological prior knowledge, whereas PGx-adapted approaches – including PRS-PGx-Bayes and mtPRS-PCA – attempt to integrate established ADME gene knowledge, pathway-level information, and multi-trait covariance structure to better reflect drug-specific biology [36, 37].

Collectively, these considerations underscore that PRS development in pharmacogenomics is not a straightforward extension of disease genetics but requires purpose-built data integration strategies aligned with the exposure-dependent nature of pharmacological phenotypes and the mechanistic principles of clinical pharmacology [38, 39].

## Current applications of polygenic risk scores in pharmacogenomics

To date, the application of PRS in pharmacogenomic research has been explored most extensively in cardiovascular medicine, where large, well-phenotyped cohorts and randomized clinical trial datasets have enabled comparatively robust analyses. In the context of lipid-lowering therapy, several studies have demonstrated that a CAD-derived PRS can stratify the magnitude of benefit from statin therapy in primary prevention. It has been shown that individuals in the highest PRS strata exhibit greater relative risk reduction than those at lower genomic risk [40, 41]; providing one of the strongest pieces of evidence to date for the clinical utility and benefit of a pharmacogenomic PRS. Similar approaches have been applied to PCSK9 inhibition, where post-hoc analyses of major outcomes trials suggest that a PRS may distinguish between absolute and relative treatment benefits, supporting a potential role for genomic risk stratification in identifying patients most likely to derive a net clinical benefit [42, 43]. Beyond lipid-lowering therapies, exploratory analyses have examined PRS-informed response to antiplatelet therapy, including clopidogrel [16], and to beta-blockers in heart failure [44], frequently employing SNP-by-treatment interaction models to identify genetic modulation of therapeutic effect independent of baseline disease risk.

In psychiatry, PRS applications have focused primarily on predicting treatment response and adverse effects, although the evidence base remains less consistent across studies. For antipsychotic pharmacotherapy, PRS derived from GWAS in schizophrenia have been investigated in relation to clozapine dosing requirements and treatment-resistant schizophrenia, findings across independent cohorts have been heterogeneous reflecting the inherent difficulty of disentangling disease genetic liability from pharmacologically relevant drug-response biology [45 – 47]. Studies evaluating PRS for antidepressant response to selective serotonin and serotonin-norepinephrine reuptake inhibitors (SSRIs/SNRIs) – including multimodal approaches integrating clinical and electroencephalographic (EEG) biomarkers– have generally yielded modest predictive performance [48, 49]. Lithium response represents one of the more mature psychiatric PRS applications. Analyses from the International Consortium on Lithium Genetics (ConLiGen) have demonstrated statistically significant associations, though with limited explained variance (approximately 2 – 3 %; [50]). More recent multimodal approaches combining PRS with clinical and demographic factors have shown improved predictive performance for lithium response [51], suggesting that integrated models may better capture the compelex genetic architecture underlying treatment outcomes while also illustrating both the promise and current ceiling of polygenic prediction in neuropsychiatric pharmacotherapy.

Beyond cardiovascular and psychiatric medicine, emerging investigations are exploring PRS-guided strategies in oncology – particularly for the prediction of chemotherapy toxicity and response to targeted molecular therapies [52]. This is highly relevant given the substantial inter-individual variability in cancer treatment tolerability beyond established monogenic predictors such as *DPYD* for fluoropyrimidines and *UGT1A1* for irinotecan. Clinically important toxicities such as taxane-induced peripheral neuropthy, platinum-associated ototoxicity, and anthracycline-related cardiotoxicity likely involve broader polygenic contributions that are not captured by single-gene testing alone. Early studies suggest that PRS-based models may contribute to impoved toxicity stratification in these contexts, although prospective validation and standardized phenotype definition remain limited—for example, a PRS derived from genome-wide loci was correlated with paclitaxel-induced peripheral neuropathy severity in a chemotherapy-exposed cohort, illustrating the potential utility of germline polygenic prediction for adverse drug reactions [53]. In pain medicine, polygenic models are also being applied to characterize inter-individual variability in opioid efficacy and chronic pain trajectories [54].

Collectively, the existing literature indicates that PGx-PRS research is transitioning from proof-of-concept studies towards disease-specific implementation scenarios. Nevertheless, the field continues to be characterized by heterogeneous study designs, modest effect sizes, and limited prospective validation. These limitations reinforce the need for integrative models that combine genome-wide polygenic scores with established monogenic pharmacogenetic markers and detailed clinical phenotyping. In particular, such frameworks should explicitly incorporate rare high-impact loss-of-function or decreased-function variants in key ADME genes (e.g., *DPYD*, *TPMT*, *CYP2D6*, *CYP2C19*), which may individually exert larger effects than genome-wide PRS but are often not adequately captures by conventional GWAS-based approaches.

## The route to clinical implementation

The translation of PRS into routine pharmacogenetic and clinical practice will require several coordinated scientific, methodological, and regulatory developments. A substantial expansion of pharmacogenomics GWAS cohorts is needed to close the persistent statistical power gap between disease genetics and pharmacogenomic studies, which still typically comprise only 1,000 to 10,000 drug-exposed individuals. Closely linked to this requirement is the inclusion of globally representative, multi-ancestry cohorts to enhance score portability and to prevent the exacerbation of pre-existing health disparities attributable to ancestral imbalances in training datasets [28, 55].

Equally important is the standardization of phenotypic definitions and outcome reporting frameworks, as drug-response endpoints such as ‘treatment response’ are currently operationalized with considerable heterogeneity with respect to timing, dosing, adherence criteria, and co-medication adjustment, thereby limiting cross-study comparability. Methodological harmonization must be coupled with improved integration of electronic health record (EHR) infrastructures to enable scalable phenotyping, real-world validation, and eventual clinical deployment, alongside systematic external validation in independent and geographically diverse cohorts [56, 57].

From a clinical translation perspective, interpretability remains a key barrier: most PGx-PRS studies report relative associations rather than absolute risk metrics, lack standardized decision thresholds for clinical application, and provide uncertain incremental predictive value over established clinical predictors. Addressing these gaps will require the development of expert consensus guidelines, PRS-specific clinical action thresholds, and demonstration of additive benefit over validated clinical risk tools by prospective outcome trials. Emerging work emphasizes integrative prediction models in which PRS are embedded within existing risk frameworks – for example, combining genomic risk scores with cardiovascular prevention tools such as QRISK [58] or heart failure prognostic indices such as MAGGIC [59] – while formally evaluating whether genetic and clinical effects are best modelled additively or multiplicatively.

Finally, successful clinical implementation will depend on transparent regulatory frameworks and clearly defined evidence standards, supported by agencies such as the EMA and FDA, to ensure analytical validity, clinical utility, and equitable deployment across patient populations.

In Germany, the application of pharmacogenetic testing, including emerging PGx-PRS approaches, must also be considered in the context of the Genetic Diagnostics Act (Gendiagnostikgesetz, GenDG), which distinguishes diagnostic from predictive genetic testing, with corresponding implications for consent procedures, medical oversight, and counseling requirements. § 3 No. 7 lit. c GenDG explicitly addresses pharmacogenetics: a genetic investigation aimed at clarifying whether genetic characteristics are present that may influence the effect of a drug is classified as a *diagnostic* genetic test. In a concrete treatment context, i.e. when the test informs the prescription of a specific drug, this classification is immediate.

Less self-evident from the statutory wording alone is the situation of preemptive pharmacogenetic testing peformed outside a specific treatment setting, for instance in the form of a pharmacogenetic passport, in which an individual is genotyped in advance for variants that may become relevant for future prescriptions. Authorative guidance on such questions of interpretation is provided by the Commission on Genetic Testing (Gendiagnostik-Kommission, GEKO), an interdisciplinary commission established under § 23 GenDG at the Robert Koch Institute, whose guidelines concretize the recognized state of science and technology in genetic teting in a legally binding manner, and which additionally publishes activity reports reflecting its ongoing assessment of developments in the field. In its activity report for the period 2022–2024 the GEKO classified preemptive pharmacogenetic testing, such as the issuing of pharmacogenetic passports, as “preemptive diagnostics” (“präemptive Diagnostik”), thereby positioning it as diagnostic rather than predictive testing under the GenDG [60]. In practical terms, this means that pharmacogenetic testing is subject to the medical practitioner reservation (Arztvorbehalt, § 7 GenDG) and to the offer of genetic counseling upon disclosure of results (§ 10 Abs. 1 GenDG) but not the more stringent requirements applicable to predictive testing under § 10 Abs. 3 GenDG.

## Future developments

Future progress in PGx-PRS will likely be driven by convergent methodological innovations and expanded clinical integration. Advances in machine learning and artificial intelligence – including deep learning architectures specifically optimized for polygenic score construction – are expected to improve the modelling of non-linear variant interactions, the contribution of rare variants, and gene-by-environment interplay that current linear models inadequately capture [61]. The integration of multi-omics layers, encompassing transcriptomics, epigenomics, proteomics, and metabolomics [62], will enable biologically informed scores that more closely reflect functional molecular consequences rather than relying solely on statistical genomic associations.

In parallel, the concept of dynamic PRS – incorporating longitudinal phenotypic and exposure data, including age, comorbidity trajectories, and cumulative drug exposure – may enable time-dependent prediction of drug response and toxicity risk. Multi-ancestry methodological frameworks will be essential to ensure cross-population transferability, with trans-ethnic fine-mapping improving causal variant resolution and population-specific calibration strategies preventing performance disparities across clinically diverse patient groups [55].

Expanded clinical applications are envisioned through integrated risk frameworks that jointly model disease susceptibility and drug-response prediction, enabling simultaneous estimation of therapeutic benefit, optimal drug selection across competing therapeutic options, and individual susceptibility to adverse drug reactions within a unified algorithmic platform. Emerging therapeutic areas – including immunotherapy, gene-based therapies, and personalized vaccination strategies – may derive particular benefit from PGx-PRS approaches given their pronounced inter-individual response variability and high per-treatment cost.

A further key direction is precision dosing: PRS-guided dose titration, integrated with pharmacokinetic/pharmacodynamic (PK/PD) modelling and therapeutic drug monitoring (TDM), could shift pharmacogenomics from categorical prescribing decisions towards continuous, individualized drug exposure optimization. Notably, TDM is already well established in clinical psychiatry (e.g., clozapine and tricyclic antidepressants) [25] and transplantation medicinve (e.g., tacrolimus and ciclosporin) [63], providing an existing infrastructure for dose optimization and longitudinal monitoring. PGx-PRS may therefore have a particularly direct translation pathway in these fields complementing established TDM frameworks with pre-treatment genomic risk stratification and individualized dose targets.

From a translational and regulatory perspective, growing discussion concerns the embedding of PGx-PRS into drug development pipelines not only as companion diagnostic tools at the marketing authorization stage, but also at earlier developmental phases to enrich trial populations, mitigate toxicity signals, and improve the probability of clinical success. The recent approval of the anti-amyloid monoclonal antibody lecanemab illustrates how genetic information can inform pharmacogenetic risk stratification. Lecanemab is approved for patients with early Alzheimer’s disease and confirmed amyloid pathology, but treatment safety is influenced by APOE ε4, the strongest common genetic risk factor for late-onset Alzheimer’s disease. In the phase III CLARITY-AD trial, ε4 carriers—particularly homozygotes—showed markedly higher rates of amyloid-related imaging abnormalities (ARIA) compared with non-carriers, while efficacy appeared broadly similar across genotype groups. This led to regulatory recommendations for pre-treatment APOE genotyping to support clinical decision-making and safety management [64].

Importantly, this case also highlights why PRS may provide added clinical value beyond monogenic stratification. Although APOE status captures a major component of ARIA susceptibility, it does not fully explain inter-individual differences in treatment response or toxicity risk. A PRS framework could integrate multiple common risk variants across relevant pathways (e.g., neuroinflammation, vascular integrity, amyloid processing), potentially enabling a more comprehensive and clinically actionable prediction models to guide patient selection and monitoring intensity.

## Conclusion

PRS shift pharmacogenetics to pharmacogenomics by capturing the polygenic architecture of drug response, treatment resistance, and adverse drug reactions more comprehensively than single-variant approaches. Early evidence – particularly from cardiovascular medicine and emerging psychiatric applications – demonstrates the potential of PGx-PRS to refine benefit–risk stratification, guide dosing decisions, and identify patients at higher likelihood of therapeutic response or drug toxicity. However, important challenges remain, including limited pharmacogenomic GWAS sample sizes, lack of methodological standardization, uncertain cross-ancestry transferability, and insufficient prospective evidence of clinical benefit. Progress will require larger multi-ancestry datasets, harmonized analytical frameworks, systematic integration with clinical and pharmacokinetic predictors, and prospective validation in clinical practice and drug development settings. With these advances, PGx-PRS carry the potential to transition from exploratory research tools into clinically actionable decision-support tools for precision prescribing.

## References

[1] Pirmohamed M (2014). Personalized pharmacogenomics: predicting efficacy and adverse drug reactions. Annu Rev Genomics Hum Genet 15.

[2] Relling M V., Evans WE (2015). Pharmacogenomics in the clinic. Nature 526.

[3] Abdullah-Koolmees H, van Keulen AM, Nijenhuis M, Deneer VHM (2021). Pharmacogenetics Guidelines: Overview and Comparison of the DPWG, CPIC, CPNDS, and RNPGx Guidelines. Front Pharmacol 11.

[4] Whirl-Carrillo M, McDonagh EM, Hebert JM (2012). Pharmacogenomics knowledge for personalized medicine. Clin Pharmacol Ther 92.

[5] Bousman CA, Bengesser SA, Aitchison KJ (2021). Review and Consensus on Pharmacogenomic Testing in Psychiatry. Pharmacopsychiatry 54.

[6] Caudle K, Klein T, Hoffman J (2014). Incorporation of pharmacogenomics into routine clinical practice: the Clinical Pharmacogenetics Implementation Consortium (CPIC) guideline development process. Curr Drug Metab 15.

[7] Amstutz U, Henricks LM, Offer SM (2018). Clinical Pharmacogenetics Implementation Consortium (CPIC) Guideline for Dihydropyrimidine Dehydrogenase Genotype and Fluoropyrimidine Dosing: 2017 Update. Clin Pharmacol Ther 103.

[8] Henricks LM, Lunenburg CATC, de Man FM (2018). DPYD genotype-guided dose individualisation of fluoropyrimidine therapy in patients with cancer: a prospective safety analysis. Lancet Oncol 19.

[9] Roncato R, Perfler S, Pasin D (2025). Uptake of DPYD and UGT1A1 testing in Italy and adherence to pharmacogenetic guidelines: A 5‐year perspective from an EQA provider. Br J Clin Pharmacol 92.

[10] Gammal RS, Court MH, Haidar CE (2016). Clinical Pharmacogenetics Implementation Consortium (CPIC) Guideline for UGT1A1 and Atazanavir Prescribing. Clin Pharmacol Ther 99.

[11] Lauschke VM, Ingelman-Sundberg M (2026). The evolving landscape of pharmacogenomics: Current achievements and future directions. Pharmacol Rev 78.

[12] Zhou K, Pearson ER (2013). Insights from genome-wide association studies of drug response. Annu. Rev. Pharmacol. Toxicol. 53.

[13] Henriksen AP, Rodríguez CL, Currant H (2025). Genome-wide associations spanning 194 in-hospital drug dosage change phenotypes highlight diverse genetic backgrounds in concurrent drug therapy. Comput Struct Biotechnol J 28.

[14] Pirmohamed M, Burnside G, Eriksson N (2013). A Randomized Trial of Genotype-Guided Dosing of Warfarin. New England Journal of Medicine 369.

[15] Johnson JA, Caudle KE, Gong L (2017). Clinical Pharmacogenetics Implementation Consortium (CPIC) Guideline for Pharmacogenetics-Guided Warfarin Dosing: 2017 Update. Clin Pharmacol Ther 102.

[16] Verma SS, Bergmeijer TO, Gong L (2020). Genomewide Association Study of Platelet Reactivity and Cardiovascular Response in Patients Treated With Clopidogrel: A Study by the International Clopidogrel Pharmacogenomics Consortium. Clin Pharmacol Ther 108.

[17] Mangravite LM, Engelhardt BE, Medina MW (2013). A statin-dependent QTL for GATM expression is associated with statin-induced myopathy. Nature 502.

[18] Carr DF, Francis B, Jorgensen AL (2019). Genomewide Association Study of Statin-Induced Myopathy in Patients Recruited Using the UK Clinical Practice Research Datalink. Clin Pharmacol Ther 106.

[19] Zhao M, Ma J, Li M (2022). Different responses to risperidone treatment in Schizophrenia: a multicenter genome-wide association and whole exome sequencing joint study. Transl Psychiatry 12.

[20] Fabbri C, Kasper S, Kautzky A (2019). Genome-wide association study of treatment-resistance in depression and meta-analysis of three independent samples. Br J Psychiatry 214.

[21] Li QS, Tian C, Hinds D (2020). Genome-wide association studies of antidepressant class response and treatment-resistant depression. Transl Psychiatry 10.

[22] Singh S, Stocco G, Theken KN (2024). Pharmacogenomics polygenic risk score: Ready or not for prime time?. Clin Transl Sci 17.

[23] Link E, Parish S, Armitage J (2008). SLCO1B1 variants and statin-induced myopathy--a genomewide study. N Engl J Med 359.

[24] Postmus I, Trompet S, Deshmukh HA (2014). Pharmacogenetic meta-analysis of genome-wide association studies of LDL cholesterol response to statins. Nat Commun 5.

[25] Hiemke C, Bergemann N, Clement HW (2018). Consensus Guidelines for Therapeutic Drug Monitoring in Neuropsychopharmacology: Update 2017. Pharmacopsychiatry 51.

[26] Daly AK (2010). Genome-wide association studies in pharmacogenomics. Nature Reviews Genetics 2010 11:4 11.

[27] McInnes G, Yee SW, Pershad Y, Altman RB (2021). Genomewide Association Studies in Pharmacogenomics. Clin Pharmacol Ther 110.

[28] Martin AR, Kanai M, Kamatani Y (2019). Clinical use of current polygenic risk scores may exacerbate health disparities. Nat Genet 51.

[29] Hicks J, Swen J, Gaedigk A (2014). Challenges in CYP2D6 phenotype assignment from genotype data: a critical assessment and call for standardization. Curr Drug Metab 15.

[30] Taylor C, Crosby I, Yip V (2020). A Review of the Important Role of CYP2D6 in Pharmacogenomics. Genes (Basel) 11.

[31] Lee M van der, Kriek M, Guchelaar HJ, Swen JJ (2020). Technologies for Pharmacogenomics: A Review. Genes 2020, Vol 11, Page 1456 11.

[32] van der Lee M, Rowell WJ, Menafra R (2021). Application of long-read sequencing to elucidate complex pharmacogenomic regions: a proof of principle. The Pharmacogenomics Journal 2021 22:1 22.

[33] Logsdon GA, Vollger MR, Eichler EE (2020). Long-read human genome sequencing and its applications. Nat Rev Genet 21.

[34] Privé F, Arbel J, Vilhjálmsson BJ (2021). LDpred2: better, faster, stronger. Bioinformatics 36.

[35] Ge T, Chen CY, Ni Y (2019). Polygenic prediction via Bayesian regression and continuous shrinkage priors. Nat Commun 10.

[36] Zhai S, Zhang H, Mehrotra D V., Shen J (2022). Pharmacogenomics polygenic risk score for drug response prediction using PRS-PGx methods. Nat Commun 13.

[37] Zhai S, Guo B, Wu B (2023). Integrating multiple traits for improving polygenic risk prediction in disease and pharmacogenomics GWAS. Brief Bioinform 24.

[38] Lewis CM, Vassos E (2020). Polygenic risk scores: from research tools to clinical instruments. Genome Med 12.

[39] Torkamani A, Wineinger NE, Topol EJ (2018). The personal and clinical utility of polygenic risk scores. Nat Rev Genet 19.

[40] Natarajan P, Young R, Stitziel NO (2017). Polygenic risk score identifies subgroup with higher burden of atherosclerosis and greater relative benefit from statin therapy in the primary prevention setting. Circulation 135.

[41] Oni-Orisan A, Haldar T, Cayabyab MAS (2022). Polygenic Risk Score and Statin Relative Risk Reduction for Primary Prevention of Myocardial Infarction in a Real-World Population. Clin Pharmacol Ther 112.

[42] Sabatine MS, Giugliano RP, Keech AC (2017). Evolocumab and Clinical Outcomes in Patients with Cardiovascular Disease. New England Journal of Medicine 376.

[43] Schwartz GG, Steg PG, Szarek M (2018). Alirocumab and Cardiovascular Outcomes after Acute Coronary Syndrome. New England Journal of Medicine 379.

[44] Lanfear DE, Luzum JA, She R (2020). Polygenic Score for Beta-Blocker Survival Benefit in European Ancestry Patients with Reduced Ejection Fraction Heart Failure. Circ Heart Fail 13.

[45] Werner MCF, Wirgenes KV, Haram M (2020). Indicated association between polygenic risk score and treatment-resistance in a naturalistic sample of patients with schizophrenia spectrum disorders. Schizophr Res 218.

[46] Wimberley T, Gasse C, Meier SM (2017). Polygenic Risk Score for Schizophrenia and Treatment-Resistant Schizophrenia. Schizophr Bull 43.

[47] Kappel DB, Legge SE, Hubbard L (2023). Genomic Stratification of Clozapine Prescription Patterns Using Schizophrenia PolygenicScores. Biol Psychiatry 93.

[48] Meijs H, Prentice A, Lin BD (2022). A polygenic-informed approach to a predictive EEG signature empowers antidepressant treatment prediction: A proof-of-concept study. European Neuropsychopharmacology 62.

[49] Serretti A, Fabbri C, Fanelli G (2025). Polygenic scores and antidepressant treatment outcomes in major depression: a critical integrative review. Neuroscience Applied 4.

[50] Amare AT, Schubert KO, Hou L (2018). Association of Polygenic Score for Schizophrenia and HLA Antigen and Inflammation Genes With Response to Lithium in Bipolar Affective Disorder: A Genome-Wide Association Study. JAMA Psychiatry 75.

[51] Cearns M, Amare AT, Schubert KO (2022). Using polygenic scores and clinical data for bipolar disorder patient stratification and lithium response prediction: machine learning approach. The British Journal of Psychiatry 220.

[52] Tran VHLJ, Paci A, Beaumais TA de, Rouleau E (2025). Pharmacogenetics in oncology: Unveiling its potential in treatment personalization and beyond. Bull Cancer 112.

[53] Hooshmand K, Goldstein D, Timmins HC (2022). Polygenic risk of paclitaxel-induced peripheral neuropathy: a genome-wide association study. J Transl Med 20.

[54] De Gregori M, Diatchenko L, Ingelmo PM (2016). Human Genetic Variability Contributes to Postoperative Morphine Consumption. J Pain 17.

[55] Wojcik GL, Graff M, Nishimura KK (2019). Genetic analyses of diverse populations improves discovery for complex traits. Nature 570.

[56] Denny JC, Ritchie MD, Basford MA (2010). PheWAS: demonstrating the feasibility of a phenome-wide scan to discover gene-disease associations. Bioinformatics 26.

[57] Sadler MC, Apostolov A, Cevallos C (2025). Leveraging large-scale biobank EHRs to enhance pharmacogenetics of cardiometabolic disease medications. Nature Communications 2025 16:1 16.

[58] Hippisley-Cox J, Coupland C, Brindle P (2017). Development and validation of QRISK3 risk prediction algorithms to estimate future risk of cardiovascular disease: prospective cohort study. BMJ 357.

[59] Pocock SJ, Ariti CA, McMurray JJV (2013). Predicting survival in heart failure: a risk score based on 39 372 patients from 30 studies. Eur Heart J 34.

[60] (205). Fünfter Tätigkeitsbericht der Gendiagnostik-Kommission. Fünfter Tätigkeitsbericht der Gendiagnostik-Kommission.

[61] Khera A V., Chaffin M, Aragam KG (2018). Genome-wide polygenic scores for common diseases identify individuals with risk equivalent to monogenic mutations. Nat Genet 50.

[62] Karczewski KJ, Snyder MP (2018). Integrative omics for health and disease. Nat Rev Genet 19.

[63] van Gelder T, Gelinck A, Meziyerh S (2024). Therapeutic drug monitoring of tacrolimus after kidney transplantation: trough concentration or area under curve‐based monitoring?. Br J Clin Pharmacol 91.

[64] van Dyck C, Swanson C, Aisen P (2023). Lecanemab in Early Alzheimer’s Disease. N Engl J Med 388.

